# A One–Two Punch to Bone: Assessing the Combined Impact of Lead and a High-Fat Diet

**DOI:** 10.1289/ehp.123-A264

**Published:** 2015-10-01

**Authors:** Julia R. Barrett

**Affiliations:** Julia R. Barrett, MS, ELS, a Madison, WI–based science writer and editor, is a member of the National Association of Science Writers and the Board of Editors in the Life Sciences.

Lead exposure and obesity each adversely affect bone formation and maintenance, which can potentially lead to low bone mass and an increased risk of fracture.^1^^,^^2^ A new study in mice, reported in this issue of *EHP*, found that lead exposure combined with a high-fat diet altered metabolic variables as well as bone quality more than either factor alone.^1^ The study also identified clues to molecular mechanisms that could explain the observed metabolic and skeletal changes.

The development and maintenance of healthy bone depend on a careful balance of formation and resorption (breakdown).^3^ Cells known as osteoblasts control bone formation, and osteoclasts manage bone resorption. Intricate networks of protein messengers regulate the numbers and activities of these cells, starting with their differentiation from precursors in the bone marrow—mesenchymal stem cells (MSCs) for osteoblasts and hematopoietic stem cells for osteoclasts.^3^ MSCs can also differentiate into adipocytes (fat cells), depending on the proteins that are present.^4^

**Figure:**
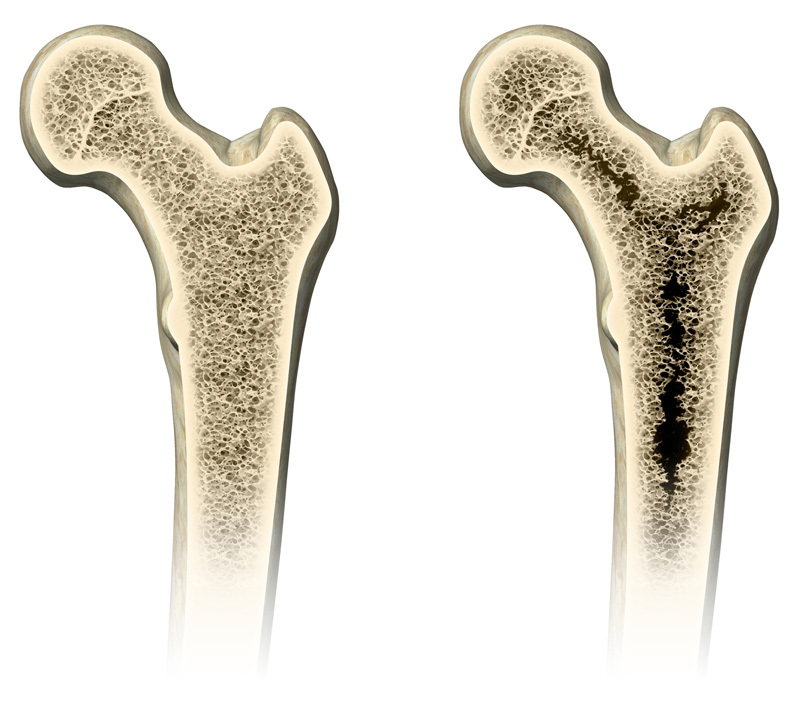
Lead exposure and obesity are both associated with increased risk of osteoporosis in humans. A new study explores potential mechanisms behind these associations. © Henning Dalhoff/Getty Images

A form of protein messaging called Wnt signaling has drawn the attention of researchers because specific bone disorders have been linked to mutation-related errors in this pathway.^5^ Subsequent research showed that disruption of Wnt signaling impairs bone health, and recent experimental studies indicated both lead and obesity cause their adverse impacts on bone at least in part through disrupted Wnt signaling.^1^^,^^4^^,^^5^^,^^6^ These findings prompted the current study, which explores how co-occurrence of obesity and lead exposure might affect bone formation and maintenance.

The authors conducted experiments in which male mice received either lead-free water or water containing 50 ppm lead. When the mice were 5 weeks old, the two groups were subdivided into groups that were fed either a high-fat or low-fat diet. After mice had been on their respective diets for 3, 6, or 12 weeks, assays were conducted to evaluate blood and bone lead levels, body fat composition, metabolic variables, bone strength and structure, and the presence and levels of markers associated with osteoblasts, osteoclasts, Wnt signaling, and adipocyte differentiation. In addition, bone marrow was assayed for osteoclast, osteoblast, and adipocyte formation. In another set of experiments, mouse osteoblast precursor cells were treated *in vitro* with a Wnt signaling pathway activator and cultured with fatty acids and/or lead. These experiments investigated the effects of these exposures on the transcription of genes that code components relevant to the Wnt signaling pathway.

At 4–8 μg/dL, the blood lead levels in the treated mice were comparable to those that occur in children in the 97.5th percentile of lead exposure.^7^ The authors also found that lead exposure and a high-fat diet were each associated with reduced bone quality in mice, which was amplified when both factors were present; however, only lead exposure was associated with statistically significant changes in bone strength. Biomarker measurements from blood and MSCs showed a shift toward bone resorption and adipocyte formation at the expense of osteoblast formation for both lead exposure and a high-fat diet. These findings suggest that the normal balance of formation and resorption in bone was tipped toward the latter and, consequently, loss of bone mass.^1^

In addition, the mice on a high-fat diet developed obesity and other symptoms of metabolic dysregulation. The *in vitro* experiments with osteoblast precursor cells indicated that both lead and fatty acids altered factors that influence Wnt signaling. This provides a potential mechanism by which obesity and lead undermine the appropriate differentiation of stem cells.^1^

“It’s an interesting study that reports an association,” says Bart Williams, director of the Center for Cancer and Cell Biology at Van Andel Research Institute in Grand Rapids, Michigan. “They’ve linked the lead exposure and a correlation with a change in some aspects of Wnt signaling. Moving forward, it will be important to really nail down the molecular mechanisms.” Williams was not involved in the study.

A particular strength of this study was that it considered more than one factor that influences bone quality. “However, there are many factors that have to be taken into account when one considers bone quality,” says coauthor Robert Mooney, a professor in the Department of Pathology and Laboratory Medicine at the University of Rochester Medical Center. “Our study was just one example of taking two factors out of the environment, putting them together, and showing that, indeed, the bone quality is poorer due to each of those contributions.”

The actual environment is enormously complex, of course, and a wide range of other factors can have an impact on bone health in humans, among them diet, exercise, and smoking.^8^ “It’s important to sort out the factors that might cause poor bone quality,” Mooney says. “Until we know, it impairs our ability to develop therapeutic approaches.”
